# Prognostic Role of Endoscopic Ultrasound Guided Direct Portal Pressure Gradient Measurement in Porto‐Sinusoidal Vascular Disorder

**DOI:** 10.1111/liv.70096

**Published:** 2025-04-19

**Authors:** Francesco Santopaolo, Lucia Giuli, Giulia Tripodi, Maria Pallozzi, Francesca Romana Ponziani, Brigida Eleonora Annicchiarico, Gianenrico Rizzatti, Andrea Contegiacomo, Alessandro Posa, Roberto Iezzi, Rosa Talerico, Antonio Gasbarrini, Alberto Larghi

**Affiliations:** ^1^ Liver Unit, CEMAD Centro Malattie Dell'apparato Digerente, Medicina Interna e Gastroenterologia Fondazione Policlinico Universitario Agostino Gemelli IRCCS Rome Italy; ^2^ Digestive Endoscopy Unit Fondazione Policlinico Universitario Agostino Gemelli IRCCS Rome Italy; ^3^ Department of Bioimaging Institute of Radiology, Fondazione Policlinico Universitario Agostino Gemelli IRCCS Rome Italy; ^4^ Department of Geriatric, Orthopedic, and Rheumatologic Sciences Fondazione Policlinico Universitario Agostino Gemelli IRCCS, Università Cattolica del Sacro Cuore Rome Italy

**Keywords:** clinically significant portal hypertension, endo‐hepatology, esophago‐gastric varices, portal pressure gradient, porto‐sinusoidal vascular disorder

## Abstract

**Background & Aims:**

HVPG is the gold standard for the diagnosis of clinically significant portal hypertension (CSPH), a condition associated with the risk of developing hepatic decompensation events. However, HVPG is an indirect method to measure portal pressure, and its application in the pre‐sinusoidal form of portal hypertension (PH), as in porto‐sinusoidal vascular disorder (PSVD), is hindered by low accuracy. Recently, endoscopic ultrasound‐guided portal pressure gradient (EUS‐PPG) measurement, which allows direct measurement of portal pressure, is emerging as a safe method and may overcome the limitation of HVPG. However, data in patients with CSPH and the pre‐sinusoidal form of PH are still missing. This study aims to evaluate the safety and usefulness of EUS‐PPG compared to HVPG in a cohort of patients with PSVD and CSPH.

**Methods:**

In this prospective single center study, patients with a diagnosis of PSVD who presented a clinical suspicion of CSPH underwent HVPG and EUS‐PPG baseline measurements. A second EUS‐PPG measurement was performed in patients naïve to non‐selective beta‐blockers (NSBBs) to evaluate haemodynamic response to therapy.

**Results:**

Twenty‐six patients were enrolled and a total of 26 HVPG and 35 EUS‐PPG measurements were performed, without any adverse events. Mean EUS‐PPG was significantly higher than mean HVPG value (16.7 ± 5.5 mmHg versus 5.5 ± 2.8 mmHg). At logistic multivariate regression analysis, EUS‐PPG value was the only variable associated with hepatic decompensation.

**Conclusions:**

EUS‐PPG measurement is safe and might have a prognostic role in patients with PSVD and CSPH, outperforming HVPG.

**Trial Registration:**

ID5486

**Abbreviations**
AEs: adverse events
CSPH: clinically significant portal hypertension
EUS‐PPG: endoscopic ultrasound‐guided portal pressure gradient
NSBBs: non‐selective beta‐blockers
PH: portal hypertension
PSVD: porto‐sinusoidal vascular disorder

**Summary**

- Porto‐sinusoidal vascular disorder (PSVD) is a rare cause of pre‐sinusoidal portal hypertension. HVPG is the gold standard for the diagnosis of portal hypertension but is hindered by low accuracy in pre‐sinusoidal forms. Endoscopic ultrasound (EUS)‐guided Portal Pressure Gradient (PPG) measurement might have a prognostic role in PSVD, outperforming HVPG.

## 1 Introduction

Portal hypertension (PH) is a hemodynamic abnormality characterised by elevated portal pressure. Regardless of its underlying cause, PH can lead to severe clinical manifestations, such as bleeding from rupture of esophago‐gastric varices, ascites, hepatic encephalopathy and hepatorenal syndrome. These conditions are associated with high morbidity and mortality rates [1]. Currently, the gold standard for diagnosing PH is the measurement of HVPG [2], which evaluates the difference between the “wedged” (or “occluded”) hepatic vein pressure and the “free” hepatic vein pressure during retrograde hepatic vein catheterization.

HVPG ≥ 10 mmHg defines clinically significant portal hypertension (CSPH), an indication to begin non‐selective beta‐blocker (NSBBs) treatment [3]. The hemodynamic response to NSBBs can be assessed through repeat HVPG measurements. While wedged hepatic vein pressure reflects hepatic sinusoidal pressure and is reliable in sinusoidal forms of PH (e.g., viral‐ and alcohol‐related cirrhosis), it may not accurately represent portal pressure in liver disease characterised by increased resistance at presinusoidal sites [4]. One such condition is porto‐sinusoidal vascular disorder (PSVD) [5], a rare cause of PH of unknown aetiology that accounts for 3%–6% of PH cases in Western countries [6].

Recently, endoscopic ultrasound‐guided portal pressure gradient (EUS‐PPG) measurement has emerged as a potential alternative, with preliminary data indicating that the technique is safe [7]. EUS‐PPG directly measures pressure values within the portal vein and hepatic veins, theoretically overcoming the limitation of HVPG in certain settings. Additionally, EUS‐PPG may provide crucial diagnostic and prognostic information regarding the severity of pre‐sinusoidal PH, as suggested by preliminary findings from our group involving a limited patient cohort [8].

We conducted a prospective study to evaluate the safety of EUS‐PPG and to assess its utility compared to HVPG in a significant cohort of patients with PSVD and CSPH.

## 2 Methods

### 2.1 Patients and Study Design

This single‐center study was conducted at Fondazione Policlinico Universitario A. Gemelli IRCSS, Rome. Between January 2023 and January 2025, we evaluated all consecutive patients diagnosed with PSVD according to the VALDIG criteria [6].

Conditions associated with PSVD have been extensively evaluated. Patients with persistent underlying conditions known to potentially reduce life expectancy were classified as having a persistent severe associated condition, as previously reported [9].

Patients with clinical suspicion of CSPH, based on the presence of esophago‐gastric varices, ectopic varices, portosystemic collaterals observed in imaging tests, and spleen stiffness measurement > 50 kPa via transient elastography, were enrolled. Exclusion criteria included portal vein thrombosis and/or a history of TIPS placement.

Eligible patients underwent baseline measurements of both HVPG and EUS‐PPG one day apart. We collected data on baseline blood tests, endoscopy results, liver and spleen stiffness measurements via transient elastography, and the history of previous episodes of hepatic decompensation (such as variceal bleeding, ascites, and hepatic encephalopathy). After PH evaluation with HVPG and EUS‐PPG, patients were followed up every three months in the outpatient clinic. In cases of hepatic decompensation, patients were hospitalised and treated according to their condition at the discretion of the primary physician.

The study protocol was approved by the ethical committee of Fondazione Policlinico Universitario A. Gemelli IRCCS, Rome, Italy on 26 January 2023 (ID 5486). All research was conducted in accordance with both the Declarations of Helsinki and Istanbul. Written consent was given in writing by all subjects.

### 2.2 Outcome Measurements

The primary objective of the study was to assess safety, defined by the incidence of adverse events (AEs). The definition and severity of AEs were determined according to the newly introduced AGREE classification for AEs in GI endoscopy [10]. Secondary objectives included: (i) comparing measurements obtained by HVPG and EUS‐PPG in this patient population; (ii) evaluating hemodynamic response to NSBBs treatment in naïve patients after titration to the maximum possible dose via repeat EUS‐PPG; (iii) determining the prognostic role of EUS‐PPG in terms of prediction of hepatic decompensation.

### 2.3 Procedures

Patients were hospitalised and underwent both procedures one day apart. HVPG measurement was performed by two expert interventional radiologists (A.C., A.P.) in accordance with the guidelines of the Baveno VII consensus [1]. All EUS‐PPG procedures were conducted under deep sedation with propofol, without orotracheal intubation, by two experienced echoendosonographers (A.L., G.R.) using a linear echoendoscope (Pentax Medical GmbH, Hamburg, Germany). The EchoTip Insight portosystemic pressure gradient measurement system (Cook Medical Europe LTD, Limerick, Ireland), which consists of a 25G needle without stylet, a connecting tube system, and a pressure transducer, was utilised for EUS‐PPG measurement.

**FIGURE 1:**
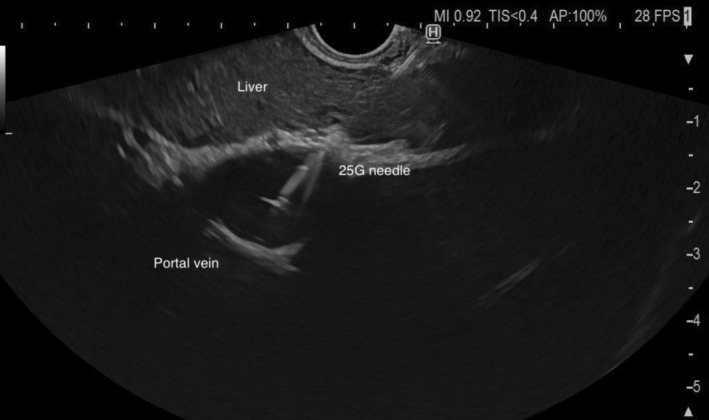
Endoscopic ultrasound‐guided measurement of portal vein pressure.

**FIGURE 2:**
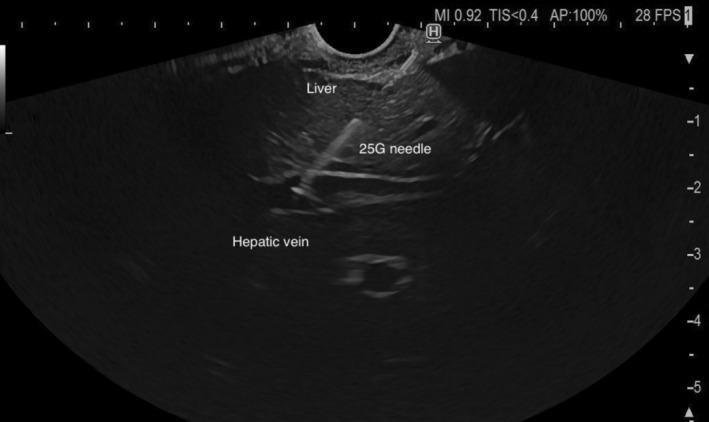
Endoscopic ultrasound‐guided measurement of hepatic vein pressure.

The needle was first primed with a heparinized saline solution to eliminate any bubbles in the tubing system. Patients were positioned supine with the manometer zeroed at the mid‐axillary line before insertion of the echoendoscope. No prophylactic antibiotics were administered prior to the puncture. Pressures were measured in the portal vein (Figure 1) and the hepatic vein (Figure 2), accessing the vessels from the stomach and traversing the hepatic parenchyma when feasible. After advancing the needle into the target vessel, this was flushed with a small amount (< 1 mL) of heparinized saline solution before each pressure reading. Each evaluation was conducted after 30 to 60 s to allow for pressure stabilisation, and three separate readings per vessel were obtained to calculate a mean pressure. Once the readings were completed, the needle was withdrawn carefully under doppler examination to ensure the absence of flow in the needle track, which would suggest active bleeding. The PPG was calculated by subtracting the hepatic vein pressure from the portal vein pressure.

## 3 Statistics

Demographic and clinical characteristics of the enrolled patients were presented as counts and percentages for categorical variables and as either mean ± SD or median (IQR) for continuous variables. Categorical variables were compared using the chi‐squared or Fisher's exact tests, while continuous variables were compared using the Student's *t*‐test or the Mann–Whitney *U* test.

The primary outcome was presented as the cumulative incidence of overall AEs in the study population. A logistic regression model was employed to determine the strength of the association between EUS‐PPG and the development of hepatic decompensation. Univariate analysis was used to estimate the association between each potential predictor and hepatic decompensation. ORs and their 95% CIs were reported. Potential predictor variables of hepatic decompensation (univariate *p*‐value < 0.05) were evaluated in the multivariate analysis, with hepatic decompensation as the dependent variable. All associations in the multivariate analysis were presented as ORs, with corresponding 95% confidence intervals and *p*‐values considered statistically significant if < 0.05. Statistical analyses were conducted using SPSS software, version 27 (IBM Corporation in Armonk, NY), and R software version 4.1.2 (CRAN, R Core 2021).

## 4 Results

During the study period, 42 patients with PSVD were identified. Sixteen patients (38.1%) were excluded due to portal vein thrombosis (5), previous TIPS placement (3), and absence of clinical or radiological signs of PH (8). Consequently, a total of 26 patients with PSVD and a suspected CSPH were enrolled in the study. Their main characteristics are reported in Table 1. All patients had preserved liver function with normal albumin and prothrombin time (PT/INR). At the time of the procedure, none of the patients were receiving anticoagulant therapy, and only one patient was on antiplatelet therapy. Seven patients (26.9%) had a severe associated condition, while 11 patients (42.3%) had a history of hepatic decompensation (seven cases of oesophageal variceal bleeding and four cases of ascites). These patients were significantly older (60.4 vs. 47.6, *p* = 0.03), had a higher mean bilirubin serum concentration (1.9 mg/dL vs. 1.1 mg/dL, *p* = 0.02) and were more frequently already on NSBBs treatment (72.7% vs. 13.3%, *p* = 0.002), compared to those without a history of liver decompensation. Nine patients were naïve to NSBBs and underwent a second EUS‐PPG measurement to evaluate hemodynamic response to treatment.

**TABLE 1:** Baseline characteristics of the 26 patients with porto‐sinusoidal vascular disorder.

Total number of patients	26
Age (yrs.)	53 ± 15.5 (yrs)
Male, *n* (%)	15 (57.7%)
Platelets ± SD (U/mL)	77.8 ± 34.1
INR ± SD	1.1 ± 0.09
Albumin ± SD (g/dL)	3.8 ± 0.4
AST ± SD (U/I)	32.5 ± 9.9
ALT ± SD (U/I)	30 ± 18.4
GGT ± SD (U/I)	63.6 ± 47
ALP ± SD (U/I)	101.3 ± 47.4
Total bilirubin ± SD (mg/dL)	1.4 ± 0.8
LSM ± SD (kPa)	7.7 ± 2.6
SSM ± SD (kPa)	71.2 ± 19.9
Number of patients on NSBBs, *n* (%)	10 (38.5%)
Number of patients with varices, *n* (%)	20 (76.9%)
Number of patients with collaterals, *n* (%)	11 (42.3%)
*Associated condition*	
No associated condition identified, *n* (%)	7 (26.9%)
Severe associated condition, *n* (%)	7 (26.9%)
Autoimmune severe	5 (19.2%)
Myasthenia gravis, *n* (%)	*n* = 2
Common variable immunodeficiency syndrome, *n* (%)	*n* = 3
Haematological severe	1 (3.85%)
Multiple myeloma, *n* (%)	*n* = 1
Human immunodeficiency virus, *n* (%)	1 (3.85%)
Mild associated condition or previously controlled associated condition, *n* (%)	12 (46.15%)
Haematological disorders	2 (7.7%)
Lymphoma (in complete remission)	*n* = 1
Selective IgA deficiency	*n* = 1
Prothrombotic disorders	1 (3.85%)
Heterozygous Prothrombin G20210A mutation	*n* = 1
Associated medications	9 (34.61%)
Azathioprine	*n* = 1
Oxaliplatin	*n* = 6
Trastuzumab	*n* = 2
HVPG ± SD (mmHg)	5.5 ± 2.8
PPG ± SD (mmHg)	16.7 ± 5.5

Abbreviations: ALP, alkaline phosphatase; ALT, alanine transaminase; AST, aspartate transaminase; GGT, gamma glutamyl transferase; INR, international normalised ratio; LSM, liver stiffness measurement; NSBBs, non‐selective beta‐blockers; PPG, portal pressure gradient; SSM, spleen stiffness measurement.

In the overall cohort, a total of 26 HVPG and 35 EUS‐PPG measurements were performed. Baseline procedures were successfully completed in all patients, with no severe or fatal AEs. Two mild episodes of oozing from the gastric wall entry site of the needle used to penetrate the portal vein or the hepatic veins during EUS‐PPG were observed; these were easily managed endoscopically with placement of an endoscopic clip. These two events were not classified as AEs according to AGREE classification, because they did not alter the standard post‐procedural course. In both cases, the EUS‐PPG procedure was completed without the need for blood transfusion and/or prolongation of the hospital stay. Of note, four EUS‐guided liver biopsies using a 19G FNA needle (Cook Medical Europe LTD, Limerick, Ireland) and three endoscopic band ligations were performed in the same session of the EUS‐PPG procedure.

The mean EUS‐PPG value detected in the 26 patients was 16.7 ± 5.5 mmHg, which was significantly higher than the mean HVPG measurement of 5.5 ± 2.8 mmHg. Regarding the HVPG procedure, hepatic venous‐to‐venous communications—which can lead to an underestimation of wedged hepatic vein pressure—were detected in four patients. However, in all cases, the hepatic vein could be successfully occluded distal to the communication.

Patients with a previous episode of hepatic decompensation had higher mean EUS‐PPG values (20.9 ± 2 vs. 13.7 ± 3 mmHg, *p* = 0.0002) compared to those without decompensation (Table 2).

**TABLE 2:** Baseline characteristics of the 26 patients with porto‐sinusoidal vascular disorder divided by the presence or absence of previous hepatic decompensation.

Parameters	No hepatic decompensation (No. 15)	Previous hepatic decompensation (No. 11)	*p*
Mean age (yrs.)	47.6 ± 14.8	60.4 ± 13.7	**0.03**
Male, *n* (%)	7 (46.7%)	8 (72.7%)	0.2
Platelets ± SD (U/mL)	86.9 ± 37.3	65.3 ± 25.6	0.1
INR ± SD	1.1 ± 0.1	1.1 ± 0.1	0.4
Albumin ± SD (g/dL)	3.8 ± 0.4	3.7 ± 0.4	0.4
AST ± SD (U/I)	31.3 ± 9.6	34.2 ± 10.5	0.5
ALT ± SD (U/I)	32.7 ± 18.1	26.9 ± 19.3	0.4
GGT ± SD (U/I)	76.6 ± 50.9	45.9 ± 36.1	0.1
ALP ± SD (U/I)	107.1 ± 46.5	93.9 (±49.7)	0.5
Total bilirubin ± SD (mg/dL)	1.1 (± 0.6)	1.9 (± 0.9)	**0.02**
LSM ± SD (kPa)	7.9 (± 2.6)	8.7 (± 2.1)	0.45
SSM ± SD (kPa)	73 ± 15.9	76.9 (± 10.8)	0.5
Number of patients on NSBBs, *n* (%)	2 (13.3%)	8 (72.7%)	**0.002**
Number of patients with varices, *n* (%)	10 (66.7%)	10 (90.9%)	0.1
Number of patients with collaterals, *n* (%)	6 (40%)	5 (45.4%)	0.8
Severe conditions associated (%)	3 (20%)	4 (36.4%)	0.3
HVPG ± SD (mmHg)	5.3 (± 3)	5.7 (± 2.8)	0.7
PPG ± SD (mmHg)	13.7 (± 3)	20.9 (± 2.6)	**0.0002**

Abbreviations: ALP, alkaline phosphatase; ALT, alanine transaminase; AST, aspartate transaminase; GGT, gamma glutamyl transferase; INR, international normalised ratio; LSM, liver stiffness measurement; NSBBs, non‐selective beta‐blockers; PPG, portal pressure gradient; SSM, spleen stiffness measurement.

Univariate logistic regression analysis identified EUS‐PPG value (OR 1.86, 95% CI 1.23–3.84, *p* = 0.025) and age (OR 1.09, 95% CI 1.01–1.22, *p* = 0.05) as significant predictors of hepatic decompensation. However, in the multivariate analysis, only EUS‐PPG remained independently associated with hepatic decompensation (*p* = 0.02) (Table 3).

**TABLE 3:** Univariate and multivariate logistic regression of risk factors associated with hepatic decompensation.

Risk factors associated with hepatic decompensation						
Parameters	Univariate			Multivariate		
	OR	95% (CI)	*p*	OR	95% (CI)	*p*
Age	1.07	1.01 to 1.16	**0.05**	1.01	0.90 to 1.15	0.79
Sex, male	3.50	0.56 to 30.7	0.20			
INR	45	0.0123 to 3 869 169	0.37			
Platelet	0.98	0.95 to 1.00	0.12			
Albumin	0.39	0.04 to 3.05	0.38			
Bilirubin	2.62	0.96 to 9.51	0.09			
HVPG	1.05	0.78 to 1.41	0.75			
PPG	2	1.33 to 4.09	**0.01**	1.94	1.25 to 4.15	**0.025**
LSM	1.21	0.89 to 1.72	0.24			
SSM	1.05	1 to 1.13	0.08			
Varices	5.00	0.65 to 105.00	0.17			
Collaterals	1.25	0.025 to 6.18	0.78			
Severe conditions associated	2.29	0.39 to 14.7	0.36			

Abbreviations: LSM, liver stiffness measurement; PPG, portal pressure gradient; SSM, spleen stiffness measurement.

Among the nine patients who were naïve to NSBBs, the mean baseline EUS‐PPG value was 17.1 ± 3.4 mmHg, which decreased to 15.1 ± 2.8 mmHg after titration of NSBBs to the maximum dose. All patients were started on carvedilol, with titration up to a maximum dose of 12.5 mg. Overall, a significant haemodynamic response, defined as a decrease in EUS‐PPG > 20% from the baseline value or an absolute value of EUS‐PPG < 12 mmHg, was achieved in only four of the nine (44.4%) treated patients. Esophago‐gastric varices were present in six of the nine patients.

After baseline evaluation, patients were followed for a mean time of 16 ± 7.7 months. Three patients experienced an episode of hepatic decompensation. One patient, with a history of variceal bleeding and already on therapy with NSBBs, developed a new bleeding episode. The other two patients, who were naïve to NSBBs and did not show a hemodynamic response, developed overt hepatic encephalopathy and ascitic decompensation, respectively.

## 5 Discussion

We conducted a study aimed at evaluating the safety of EUS‐PPG in a cohort of patients with PSVD and CSPH. Overall, we found that all 35 EUS‐PPG procedures performed were highly safe. We prioritised safety as the first outcome since this study focused on patients with PSVD and associated CSPH. Indeed, this patient group is particularly vulnerable to bleeding for two main reasons: (i) presence of varices and/or portosystemic collaterals, (ii) and thrombocytopenia. In our cohort, 76% of patients had oesophageal varices and 42% had a history of hepatic decompensation. These findings are common in this patient population, as demonstrated by the eight additional patients with PSVD who were excluded from the study due to portal vein thrombosis or previous TIPS placement. In our cohort, thrombocytopenia ranged from moderate to severe with a mean platelet count of 77 × 10^9^/L, and six out of the 26 patients had platelet values < 50 × 10^9^/L.

Overall, no AEs were observed during the 35 EUS‐PPG procedures. The two cases of oozing from the needle entry side were easily managed endoscopically and were classified as no AEs in accordance with the AGREE classification [10]. EUS‐PPG proved to be not only safe, but also highly feasible, even in the presence of moderate to severe thrombocytopenia (value as above). In this regard, our cohort differed from two recently published case series. In the first series, among 23 patients only two (8.7%) had thrombocytopenia (PLT < 150 × 10^9^/L) [11]; In the second study, half of the 83 enrolled patients had thrombocytopenia, but this was associated with a low mean EUS‐PPG value (7 mmHg), while the proportion of patients with a PPG > 10 mmHg was not reported and remains unknown [7].

A recent study evaluated 373 patients who underwent EUS‐PPG across eight US centres. The mean PPG value in patients with oesophageal varices was 11.6 mmHg, which was significantly lower than the mean value recorded in our cohort (16.7 mmHg) [12]. This further indicates that PSVD can be characterised by severe PH and reinforces the safety of the procedure in the presence of oesophageal varices.

The mean portal pressure gradient measured was significantly higher when performed under EUS guidance compared to the HVPG. This finding aligns with the clinical features of CSPH, which characterised the patients in our study, and is consistent with the pre‐sinusoidal nature of PSVD. In conditions with a pre‐sinusoidal component, HVPG, which indirectly evaluates portal pressure gradient, underestimates the actual measurement, failing to provide a definitive prognostic value.

Traditionally, liver function tests such as bilirubin, INR, and albumin have been considered prognostic factors in cirrhotic patients; however, they do not hold the same value in those with PSVD who often have preserved liver function regardless of the severity of PH. Notably, at multivariate analysis, EUS‐PPG value was the only parameter independently associated with hepatic decompensation, outperforming HVPG.

Our results are in line with a recent multicenter study on the natural history of PSVD with CSPH [13], which demonstrated that HVPG does not enhance the predictive accuracy of a clinical‐biochemical prognostic model. This further reinforces the hypothesis that EUS‐PPG may have superior prognostic value compared to HVPG in these patients, because of the pre‐sinusoidal nature of their PH. Notably, EUS‐PPG may become a valuable marker for identifying patients at higher risk of disease progression, providing a more accurate assessment of hemodynamic status and aiding in risk stratification.

Based on the results of the present study, EUS‐PPG can also play a pivotal role in patients with MAFLD‐related cirrhosis, as approximately 30% of them exhibit a pre‐sinusoidal component [14]. In these cases, similar to PSVD, HVPG underestimates the portal pressure gradient, making EUS‐PPG a potential first‐line approach for evaluating PH in this patient population. Studies comparing EUS‐PPG and HVPG in this context are crucial, especially considering that MAFLD‐related cirrhosis is becoming an increasingly prevalent cause of PH.

Among the nine patients who underwent maximum titration of NSBBs treatment, repeat EUS‐PPG demonstrated a hemodynamic response in only four (44.4%) of them based on criteria validated for HVPG [15]. However, it remains unclear whether these criteria can be applied to PSVD, irrespective of the technique utilised to measure portal pressure gradient. Therefore, larger multicenter studies with a longer follow‐up period are needed to fully explore the hemodynamic response in pre‐sinusoidal forms of PH, utilising the EUS‐PPG technique. The availability of these data can theoretically help to decide the best management strategy to be recommended for PSVD patients who do not exhibit a hemodynamic response to NSBBs.

The EUS‐PPG procedure was performed in all patients under deep sedation using propofol, which is known to potentially affect HVPG measurements, unlike low doses of midazolam. The influence of deep sedation on EUS‐PPG remains to be fully defined, and studies comparing propofol with low doses of midazolam are ongoing.

One major limitation of our study is that it was conducted as a single‐center investigation within a tertiary care facility. Consequently, the results obtained might not be applicable to all centers, especially small hospital facilities, where procedural volume can be low, with a limited technical experience that can result in an increased rate of AEs. However, differently from HVPG that has never reached a widespread dissemination due to the difficulty in performing a proper measurement of the pressure gradient [1], EUS‐PPG is rapidly expanding, with a 30% increase in the number of performing centers over the past two years (unpublished data kindly provided by Cook Medical). Moreover, procedural time significantly decreases to approximately 15 min after just 10 procedures [16].

In conclusion, in patients with PSVD and CSPH, EUS‐PPG is highly safe and technically feasible, with a superior performance compared to HVPG. In addition, EUS‐PPG may have prognostic implications, which warrant further investigation in larger patient populations through multicenter studies. Our study paves the way for the utilisation of EUS‐PPG as a primary tool to evaluate the presence of PH in liver diseases characterised by the presence of a pre‐sinusoidal component.

## Author Contributions

Study concept and design: Francesco Santopaolo and Alberto Larghi. Acquisition of data: Francesco Santopaolo, Lucia Giuli, Gianenrico Rizzatti, Maria Pallozzi, Francesca Romana Ponziani, Brigida Eleonora Annicchiarico, Giulia Tripodi, Andrea Contegiacomo, Alessandro Posa, Roberto Iezzi, Alberto Larghi. Statistical analysis: Francesco Santopaolo and Rosa Talerico. Drafting the manuscript: Francesco Santopaolo and Alberto Larghi. Critical revision of manuscript: all of the authors.

## Ethics Statement

The study protocol was approved by the ethical committee of Fondazione Policlinico Universitario A. Gemelli IRCCS, Rome, Italy on 26 January 2023 (ID 5486). All research was conducted in accordance with both the Declarations of Helsinki and Istanbul. Written consent was given in writing by all subjects.

## Conflicts of Interest

The authors declare no conflicts of interest.

## Data Availability

The data that support the findings of this study are available from the corresponding author upon reasonable request.
